# Supplementary material for: Therapist perceptions of the Danish Physiotherapy Research Database for assessing patients with chronic disease
Source: PLoS One. 2021 Nov 4;16(11):e0259355. doi: 10.1371/journal.pone.0259355 (PMC8568098; doi:10.1371/journal.pone.0259355)
Supplement: S1 Table — The English version of the survey questions in relation to determining physiotherapists experiences when using the PhysDB-FCP. Some domains are modified on the basis of the framework by Huijg et al. [15]. (DOCX) [file pone.0259355.s001.docx]

**Supporting Information. S1 Table**

| **Variable** | **Description or wording of variable** | **Response options** |
| --- | --- | --- |
| Consent |  | Yes \| No |
| **General information on physiotherapists** | | |
| Sex | Are you male or female? | Female \| Male |
| Age | What is your age? | Free text |
| Experience | How many years have you practiced physiotherapy? | Free text |
| Education | Have you studied courses other than your physiotherapy degree? | Yes \| No |
|  | (If yes) What was the level of the course? | Diploma \| Masters \| Candidate \| PhD |
| Role | What is your role in the clinic? | Owner \| Manager \| Employee \| Self-employed \| Other (free text) |
| Role (FCP) | Do you treat patients with free of charge physiotherapy? | Yes No (Survey ends) |
| Patient numbers | How many free of charge physiotherapy patients have you examined using the PhysDB-FCP? | 0 \| 1-5 \| 6-10 \| 11-15 \| 16-20 \| >21 |
| Other tools | Do you use standardised tools other than the PhysDB-FCP to examine patients receiving FCP? | Yes \| No |
|  | (If yes) Which tools do you use? | Free text |
| **Physiotherapist experiences with the PhysDB-FCP** | | |
| *When using the PhysDB-FCP for examination, goal setting and evaluation in my patients receiving free of charge physiotherapy it is my experience that…* | | |
| Initial patient questionnaire | …the initial patient questionnaire was useful | Strongly Agree – Strongly Disagree |
| Baseline assessment | …the baseline assessment was useful | Strongly Agree – Strongly Disagree |
| Goal setting | …it was helpful when goal setting | Strongly Agree – Strongly Disagree |
| Change in function over time | …it was useful to assess change of function overtime | Strongly Agree – Strongly Disagree |
|  | …it was helpful to have the patient assessment and treatment data generated automatically | Strongly Agree – Strongly Disagree |
| *Other questions related to physiotherapist experiences* | | |
| Purpose of PhysDB-FCP | The purpose of PhysDB-FCP was clearly defined for me | Strongly Agree – Strongly Disagree |
| Future use of PhysDB-FCP | Is a tool like the PhysDB-FCP something you would consider using in the future? | Yes \| No \| Maybe \| Don’t know |
| Time to perform baseline assessment | I can typically perform a baseline assessment (patient questionnaire collection, patient history, goal setting and functional tests) using PhysDB-FCP in… | <45 min \| 45-60 min \| 60-75 min \| >75 min |
| Need to split baseline assessment | I divide the baseline assessment (patient questionnaire, patient history, goal setting and function tests) into multiple consultations | Always \| Often \| Sometimes \| Rarely \| Never |
| Time to perform functional tests | I can typically perform the functional tests within the PhysDB-FCP in… | < 15 min \| 15-30 min \| 30-45 min \| >45 min |
| **Physiotherapist perceptions of patient experiences** | | |
| *My perception of patients' experience with PhysDB-FCP is that…* | | |
| Patient positivity | …patients were positive about being assessed with PhysDB-FCP. | Strongly Agree – Strongly Disagree |
| Patient involvement in treatment | …patients were more involved in their physiotherapy treatment when I used the tools provided in the PhysDB-FCP | Strongly Agree – Strongly Disagree |
| **Resources and support** | | |
| Received initial training? | I was taught how to use the PhysDB-FCP by the project group behind the PhysDB-FCP | Yes \| No \| Other |
| Usefulness of initial training | I received enough training to be able to use the PhysDB-FCP | Strongly Agree – Strongly Disagree |
| Ongoing support | I have received sufficient ongoing support from the PhysDB-FCP project group | Strongly Agree – Strongly Disagree |
| Usefulness of written tutorials | The written tutorials for the amnestic section of the PhysDB-FCP were helpful | Strongly Agree – Strongly Disagree |
| Organizational resources | In the clinic where I work, all necessary resources were available for me to use the tools within the PhysDB-FCP. | Strongly Agree – Strongly Disagree |
| Organizational support | The owner/s of the clinic have supported the use of the PhysDB-FCP | Strongly Agree – Strongly Disagree |
| **Ideal frequency of PhysDB-FCP use** | | |
| Frequency of baseline assessment | How often do you think it is relevant to perform the baseline assessment using a tool such as the PhysDB-FCP | Every 6 months \| Every year \| Other (free text) |
| Frequency of functional tests | How often do you think it is relevant to perform the functional assessments using a tool such as the PhysDB-FCP | Every 3 months \| Every 6 months \| Other (free text) |
| **Open-ended question** | | |
|  | Do you have any suggestions for changes or have you experienced anything that could make PhysDB-FCP a better assessment and evaluation tool? | Free text |
