# Supplementary material for: Therapist perceptions of the Danish Physiotherapy Research Database for assessing patients with chronic disease
Source: PLoS One. 2021 Nov 4;16(11):e0259355. doi: 10.1371/journal.pone.0259355 (PMC8568098; doi:10.1371/journal.pone.0259355)
Supplement: Attachment — Submitted filename: Response to Reviewers.docx [file pone.0259355.s004.docx]

**Reply to reviewers**

Based on the reviewer comments 2, we have made amendments to the manuscript.

In the tracked changes document, yellow denotes insertions from the original submission and turquoise with a strike through indicates deletions from the original submission.

**Reviewer 1:**

**Thanks for attempting to address my comments.**
Thank you for the time taken to make the comments – We understand that peer review is voluntary and we appreciate the time it must have taken you to read, comment and (subsequently) improve our manuscript.

**Line 79, can the authors change aren't to are not**
We have changed this

**Instead of using tables, can the authors describe Table 2 and Table 3 (because they can easily be described). I made this suggestion because the Tables are scanty so are the sections that the authors refer the reader to.**
Thank you for this suggestion. As suggested, we have placed the information from the tables in the text.

**Reviewer 2:
Thanks to authors for a detailed paragraph addressing initial concerns. I have no further comments on this paper.**
Thank you
